# Fecal short chain fatty acids in children living on farms and a link between valeric acid and protection from eczema

**DOI:** 10.1038/s41598-020-79737-6

**Published:** 2020-12-31

**Authors:** Monica Gio-Batta, Fei Sjöberg, Karin Jonsson, Malin Barman, Anna-Carin Lundell, Ingegerd Adlerberth, Bill Hesselmar, Ann-Sofie Sandberg, Agnes E. Wold

**Affiliations:** 1 Department of Infectious Diseases, Institute of Biomedicine, University of Gothenburg, Guldhedsgatan 10A, 413 46 Gothenburg, Sweden; 2 Food and Nutrition Science, Department of Biology and Biological Engineering, Chalmers University of Technology, Gothenburg, Sweden; 3 Unit of Metals and Health, Institute of Environmental Medicine, Karolinska Institutet, Stockholm, Sweden; 4 Department of Rheumatology and Inflammation Research, Institute of Medicine, University of Gothenburg, Gothenburg, Sweden; 5 Department of Paediatrics, Institute of Clinical Sciences, University of Gothenburg, Gothenburg, Sweden

**Keywords:** Epidemiology, Paediatric research, Biomarkers

## Abstract

Children growing up on farms have low rates of allergy, but the mechanism for this protective effect has not been fully elucidated. Short chain fatty acids (SCFAs) produced by the gut microbiota may play a role in protection from allergy. We measured fecal SCFA levels in samples collected from 28 farming and 37 control children over the first 3 years of life using gas chromatography. Data on diet and other host factors were recorded and allergy was diagnosed at 8 years of age. Among all children, median propionic and butyric acid concentration increased over the first 3 years, and longer SCFAs typically appeared by 1 year of age. Farm children had higher levels of iso-butyric, iso-valeric and valeric acid at 3 years of age than rural controls. In addition, children with elder siblings had higher levels of valeric acid at 3 years of age, and dietary factors also affected SCFA pattern. High levels of valeric acid at 3 years of age were associated with low rate of eczema at 8 years of age. The fecal SCFA pattern in farm children suggests a more rapid maturation of the gut microbiota. Valeric acid or associated microbes may have protective potential against eczema.

## Introduction

Living on a farm is protective against immunoregulatory and inflammatory diseases, including allergic diseases^1,2^ and inflammatory bowel disease (IBD)^3^. Farmers also have reduced rates of colorectal and other cancers^4^. The prevalence of allergy and IBD has increased dramatically over the last century in industrialized countries^5,6^, while rates in farming families have remained low by comparison^1,3^. Which exposures in the farming environment that exert the protective effect is unknown, but exposure to a wider range of microbes during infancy^7^, or typical dietary patterns in farming families^8^ may contribute.

Short chain fatty acids (SCFAs) are the most abundant bacterial metabolites in the colon and have been proposed as key mediators in microbiota-induced effects on the host^9^. They contain between two and six carbon atoms, and are produced when dietary fibre and protein, mucous and sloughed epithelial cells are metabolized by gut bacteria. The major SCFAs generated in the colon are short unbranched varieties such as acetic, propionic and butyric acid. As the microbiota becomes more complex, longer SCFAs, such as valeric acid, are produced. Further, the branched SCFAs, iso-butyric and iso-valeric acid, appear. These are generated by the bacterial metabolism of protein^10^. Iso-caproic acid is a branched SCFA considered a marker of colonization by *Clostridium difficile*^11^, an obligate anaerobe that is fairly common in the gut microbiota of young children^12^.

Many SCFAs exhibit effects that could play a role in immunoregulatory and inflammatory diseases. Within the gut, SCFAs maintain epithelial barrier function by decreasing intestinal tight junction permeability^13^ and inducing secretion of the epithelium’s protective mucous layer^14^. Butyric acid is the major source of energy for colonocytes^15^ and is also a histone deacetylase inhibitor, making it important in the control of gene expression^16^. While butyrate is almost fully consumed in the colon, acetic and propionic acids can be detected in peripheral blood, where they may elicit physiological effects in various body tissues^9,17^, e.g. via activation of G-protein coupled receptors, found on the surface of many different cell types^9^. Very little is known regarding the immunomodulatory effects of longer and branched SCFAs. However, in a birth cohort study, higher levels of iso-butyric, valeric and iso-valeric acid at 1 year of age were inversely related to the development of food allergy by age four^18^.

The aim of this study was to investigate whether children growing up on small dairy farms had an altered fecal SCFA pattern compared to children living in the same rural area but not on farms, and if so to investigate whether this pattern was related to subsequent allergy outcomes. A secondary aim was to describe the development in fecal SCFA pattern over the first 3 years of life.

## Methods

### Subjects

The FARMFLORA birth cohort comprised 28 infants raised on small family-owned dairy farms in Skaraborg County in South-West Sweden and 37 infants from the same rural area but not raised on farms who were followed until 8 years of age. Pregnant women were recruited between September 2005 and May 2008. Healthy children born at term were included, and subjects from dairy farms with infrequent animal contact, from non-dairy farms, or from urban areas were excluded. The study conforms to the standards of the Declaration of Helsinki and was approved by the Regional Ethics Committee in Gothenburg (No. 363-05). Written informed consent was obtained from the parents of all participants.

The cohort characteristics are shown in Table 1. There were fewer boys in the farm group than in the rural control group (36% vs. 62%, *P* = 0.04), and farm children more often had a cat or dog (75% vs. 51%, *P* = 0.054). Farm children consumed more full-fat milk products and less margarine and oils compared with the rural control children, as reported previously^8,19^. At 1 year of age, farm children also consumed more oily fish (*P* = 0.02) and farm milk (*P* < 0.01), while control children consumed more poultry (*P* = 0.03)^8,19^.

**Table 1:** General and nutritional characteristics of farm and control children.

	Farm (n = 28)		Control (n = 37)		*P*
**Family**					
Allergic heredity, mother^a^	7 (25%)		11 (30%)		0.68
Allergic heredity, father^a^	1 (4%)		12 (32%)		0.01*
Level of education, mother^b^	2 (1–5)		4 (1–5)		0.20
Level of education, father^b^	2 (1–5)		2 (1–5)		0.02*
Smoking, mother^c^	0 (0%)		1 (3%)		1.00
Smoking, father^c^	1 (4%)		4 (11%)		0.38
Elder sibling(s)	18 (64%)		17 (46%)		0.15
Cat or dog in house^d^	21 (75%)		19 (51%)		0.05
**Birth and child**					
Mother’s age at delivery (years)	33 (21–42)		32 (22–41)		0.46
Caesarian section	3 (11%)		7 (19%)		0.50
Male	10 (36%)		23 (62%)		0.04*
Allergic at 3 years^e^	1 (4%)		10 (32%)		0.02*
Allergic at 8 years^e^	3 (11%)		7 (19%)		0.72
**Breastfeeding and child’s diet at 1 year**					
Exclusive breastfeeding (months)	4.0 (0.0–6.0)		3.5 (0.0–6.0)		0.11
Fat (g/day)	33 (7–78)		32 (14–62)		0.70
Margarine and oil (g/day)	3 (0–19)		5 (0–26)		0.10
Cream (g/day)	1 (0–28)		0 (0–14)		0.02*
Milk fats (g/day)	11 (0–35)		5 (0–29)		0.04*
Oily fish (g/day)	0 (0–63)		0 (0–15)		0.02*
Protein (g/day)	35 (5–60)		34 (19–62)		0.69
Poultry (g/day)	0 (0–5)		0 (0–75)		0.03*
Carbohydrates (g/day)	130 (23–183)		135 (65–194)		0.11
Fibre (g/day)	11 (2–18)		11 (4–18)		0.93
Fruit and vegetables (g/day)	150 (77–200)		180 (90–240)		0.22
Farm milk (g/day)^f^	0(0–343)		0 (0–0)		< 0.01*
Homemade porridge (g/day)	0 (0–175)		0 (0–100)		0.06

Data are n (%) or median (range).

*P < 0.05, Chi-squared or Fisher’s exact test for counts, Mann–Whitney U-test for continuous variables.

^a^Doctor-diagnosed asthma, rhinitis, or atopic eczema.

^b^1 = Elementary school, 2 = upper secondary school 2–3 years or equivalent, 3 = qualified graduate from upper secondary school, 4 = university < 1 year, 5 = university > 1 year.

^c^During the last month of pregnancy.

^d^At the time of recruitment.

^e^Doctor-diagnosed asthma, atopic eczema, allergic rhino-conjunctivitis or food allergy. At 3 years, n = 27 farm children and 36 rural controls; at 8 years, n = 18 farm children and 30 rural controls.

^f^Pasteurized and unpasteurized. Data selected from a previous study on the FARMFLORA birth cohort^8^, with intake of milk fats calculated here.

### Clinical examination

Children in the FARMFLORA study were examined by study pediatricians to diagnose eczema, asthma, allergic rhino-conjunctivitis and food allergy according to predefined protocols at 18 months, 3 years and 8 years of age. All diagnoses were confirmed by a specialist pediatric allergologist (BH). As described previously, living on a farm was associated with protection from allergy at 3 years of age^8^, but by 8 years of age there was no such association^20^. In brief, at 8 years of age, eczema was diagnosed according to William’s criteria, or as having recurring itchy spots on typical locations over at least 6 months, with symptoms during the last 12 months. Asthma was diagnosed based on wheeze or heavy breathing together with response to anti-inflammatory maintenance therapy, bronchial hyperresponsiveness on methacholine challenge (PD20 < 0.6 mg) or bronchial obstruction reversible to β_2_-agonist (FEV1rev ≥ 12%). Food allergy was defined as symptoms of food allergy supported by either planned or accidental open food challenge. Allergic rhino-conjunctivitis was defined as eye and/or nasal symptoms on exposure to pollen or animals together with a positive allergen-specific IgE test or skin prick test against a corresponding allergen.

Specific IgE was measured in venous blood against food (milk, egg, soy, fish, wheat and peanut; six-mix food test) and inhalant allergens (birch, timothy grass, mugwort, cat, dog, horse and house dust mite; Phadiatop), followed by specific IgE tests by ImmunoCAP (all from Phadia/Pharmacia Diagnostics, Uppsala, Sweden). An allergen-specific IgE level ≥ 0.35 kU/L was considered positive. Skin prick tests were performed in accordance with European guidelines using standard allergen extracts (birch, grass, mugwort, cat, dog, horse, rabbit, *Dermatophagoides pteronyssinus*, *Dermatophagoides farinae* and *Cladosporium* (Soluprick SQ; ALK Abello AS, Hørsholm, Denmark), as previously described^20^.

### SCFA analysis

Fecal samples were collected at regular intervals by parents and transported in gas-tight sachets to the laboratory at Gothenburg University, where they were stored frozen at − 80 °C until further analysis. Samples obtained when the children were 4 weeks, 1 year and 3 years of age were selected for analysis of the concentration of acetic, propionic, iso-butyric, butyric, iso-valeric, valeric, iso-caproic and caproic acids by gas chromatography, using a method modified from Zhao et al*.*^21^. In brief, samples were thawed at room temperature, then the feces (200 mg) was mixed with water (1 mL) and homogenized for 20 s. The suspension was adjusted to pH 2–3 using dilute hydrochloric acid, kept at room temperature for 10 min, and centrifuged for 20 min at 5000 rpm. 180 μL of the supernatant was mixed with internal standard solution (20 μL; 7.9 mM 2-ethylbutyric acid in 12% v/v aqueous formic acid). A gas chromatograph with flame ionization detector, fitted with a fused silica 30 m × 0.53 mm capillary column with free fatty acid stationary phase and with glass wool in the injection port liner, was used for analysis. The helium pressure was 30 kPa, injector temperature 200 °C, detector temperature 210 °C and injection volume 1 μL. The oven program was: 1 min at 100 °C, ramp to 170 °C at 10 °C/min, ramp to 220 °C at 20 °C/min, 4 min at 220 °C. Peaks were identified using retention time. The SCFA:internal standard peak area ratio was determined for each SCFA peak, and the corresponding SCFA concentration determined from calibration curves. SCFA levels were not determined for 18 (28%) children at 4 weeks, 1 (2%) at 1 year and 13 (20%) at 3 years of age, due to lack of material. Variables for analysis were the prevalence and concentration of each SCFA.

### Dietary assessment

Data regarding the diet of the FARMFLORA children, including full details of the dietary assessment methodology, have been published previously^8,19^. In brief, parents continuously recorded breastfeeding and formula practices, and introduction of new foods, in diaries up to 18 months of age. At 1 year of age (10–14 months), parents completed an unannounced 24-h dietary recall, followed by a 24-h food diary, of their children’s diet^8^. The collected dietary information was registered and calculated based on the food composition database of the Swedish National Food Agency using the software Dietist Net Pro (Kost och Näringsdata, version 15.11.02, Stockholm, Sweden).

### Statistical methods

The Mann–Whitney U test was used for the evaluation of differences in continuous variables between groups, as SCFA concentration and other variables were typically not normally distributed. For categorical variables the Chi-Squared test was used, or the Fisher’s Exact test for small samples. Friedman tests were used to compare the concentration of fecal SCFAs at different time points.

Multiple logistic regression was used to confirm associations between farm residence and SCFA levels after adjusting for covariates, and to identify explanatory variables. Dichotomous variables for high and low SCFA concentration were created. Variables that were associated with farm residence, and also predictive of SCFA level in univariate logistic regression analysis, (*P* ≤ 0.2 for both) were included as covariates. In addition, elder siblings and dietary intakes of protein and fibre were also included, as they have been linked theoretically with SCFA levels^9,18^. Models were built using a backward conditional method.

Statistical analyses were performed with SPSS Statistics version 25 (IBM Corporation, New York, USA). Two-tailed *P*-values of < 0.05 were considered significant.

## Results

### Maturation of fecal SCFA pattern during early childhood in cohort children

**Figure 1:**
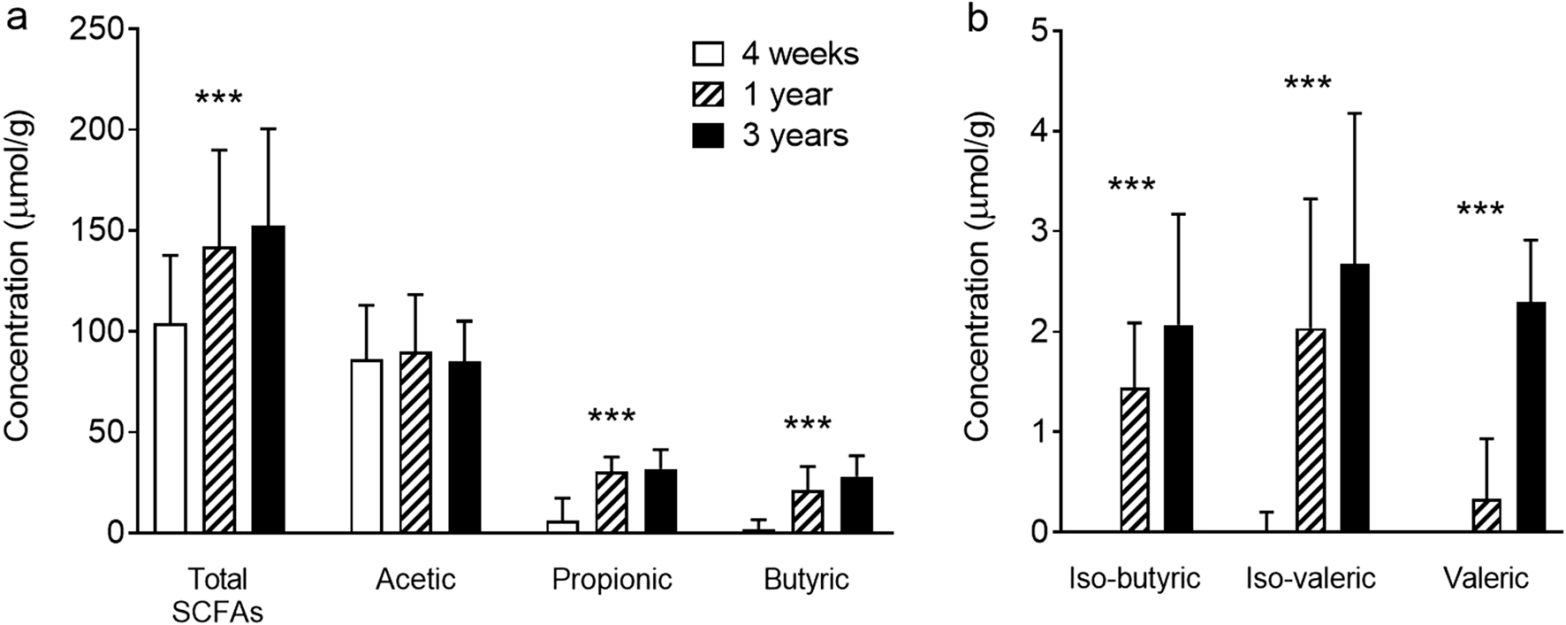
Development of fecal SCFA levels during early childhood. Median concentration of (**a**) total SCFAs and acetic, propionic and butyric acid, and (**b**) iso-butyric, iso-valeric and valeric acid, in children at 4 weeks (n = 47), 1 year (n = 64) and 3 years of age (n = 51). Error bars show the interquartile range. ***P < 0.001, Friedman test.

We first examined how fecal SCFA patterns developed during early childhood, from 4 weeks to 3 years of age, among the cohort as a whole. There was considerable variation in both the prevalence and concentration of the various SCFAs between children at any given time. Nonetheless, a notable transition was observed in SCFA patterns over the first 3 years of life (Fig. 1).

Short chain fatty acids are made up of between two and six carbon atoms (C_2_–C_6_), and may be straight-chain or branched (iso-, or *i*). At 4 weeks of age the shortest SCFA, acetic acid (C_2_), was present in all infants with a median concentration of 86 μmol per gram of feces, and represented > 80% of the total SCFA concentration. Propionic (C_3_) and butyric (C_4_) acids were each detected in at least 70% of infants, at much smaller median concentrations (6.1 and 1.9 μmol/g respectively, Fig. 1a). Iso-butyric (*i*C_4_) and iso-valeric (*i*C_5_) acids were also represented in up to 28% of infants, at concentrations up to 4 μmol/g. Finally, valeric (C_5_), caproic (C_6_) and/or iso-caproic (*i*C_6_) acids were detected in a small minority of infants, at concentrations up to 9.8, 1.8 and 1.7 μmol/g, respectively.

With age, the median total concentration of fecal SCFAs increased, from 104 μmol/g at 4 weeks of age to 152 μmol/g at 3 years of age. By 3 years of age, acetic, propionic and butyric acids were present in all children, at median concentrations of 85, 32 and 28 μmol/g, respectively (Fig. 1a). Iso-butyric, iso-valeric and valeric acids were also detected in almost all children, although at much lower median concentrations (2–3 μmol/g; Fig. 1b: note different scale than in Fig. 1a). Further, caproic acid was detected in a third of children, at concentrations up to 2 μmol/g, while iso-caproic acid was detected in just one child in twenty, at concentrations up to 0.3 μmol/g.

### Fecal SCFA patterns in farm and control children

**Figure 2:**
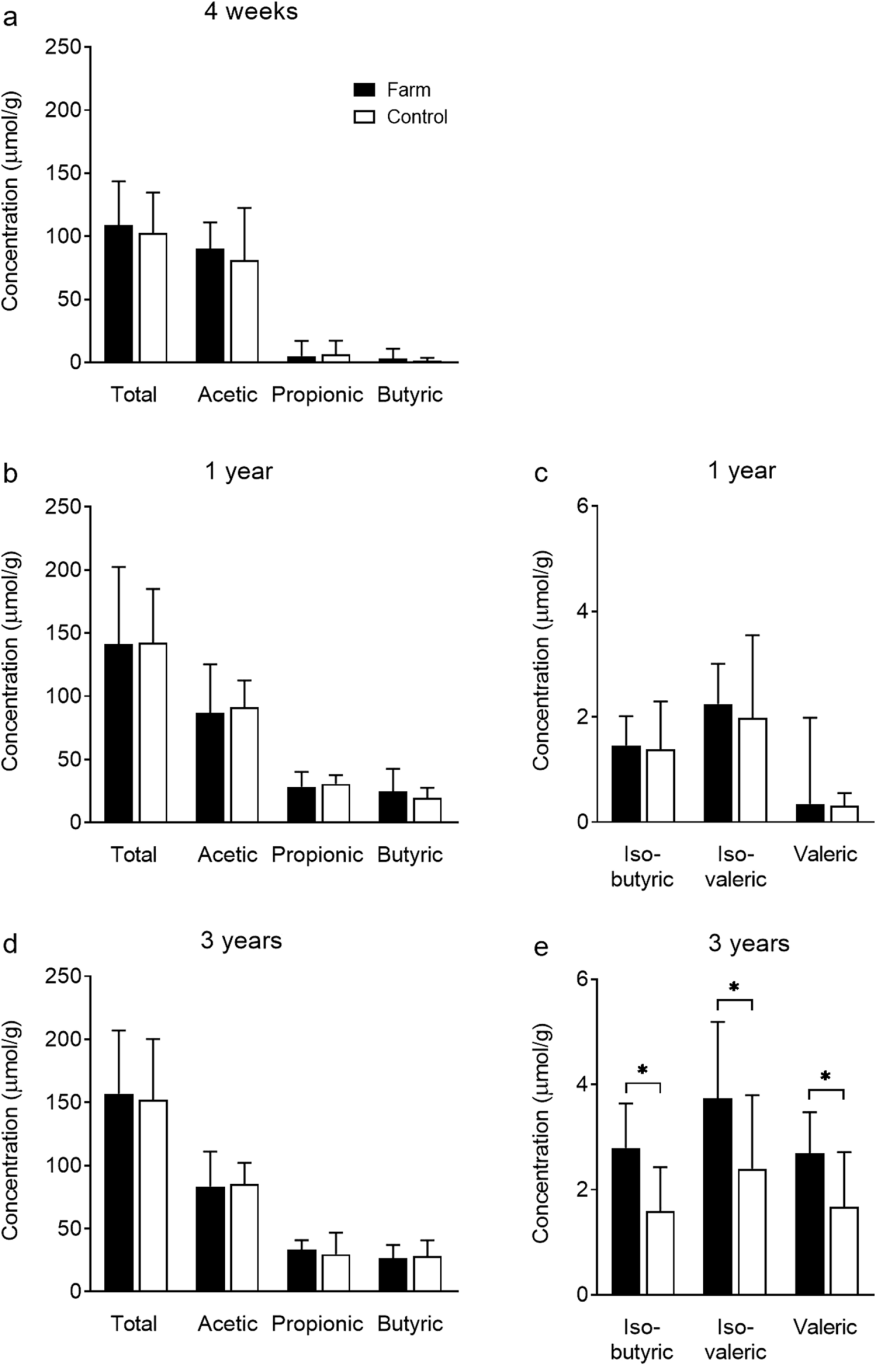
Fecal SCFA levels in farm and control children. Median concentration of total SCFAs and individual species of SCFAs in farm and rural control children at (**a**) 4 weeks, n = 24 (farm) and 23 (control); (**b**,**c**) 1 year, n = 28 (farm) and 36 (control), and (**d**,**e**) 3 years of age, n = 19 (farm) and 32 (control). Error bars show the interquartile range. *P < 0.05, Mann–Whitney U-test.

At 4 weeks and 1 year of age, no significant differences were observed in the concentration of fecal SCFAs in children living on farms, compared to children living in the same rural area but not on farms (Table 2, Fig. 2a–c). However, at 3 years of age (Fig. 2d,e), children living on farms had higher median concentrations of several fecal SCFAs including iso-butyric (2.8 vs. 1.6 μmol/g, *P* = 0.03), iso-valeric (3.7 vs. 2.4 μmol/g, *P* = 0.03) and valeric acid (2.7 vs. 1.7 μmol/g, *P* = 0.01) than control children.

**Table 2:** Prevalence and concentration of fecal SCFAs in farm and control children.

	Prevalence n (%)		Concentration, μmol/g Median (range)			
	Farm	Control	Farm		Control	
**4 weeks**^a^						
Total SCFAs	–	–	109 (42–220)		103 (29–269)	
Acetic	24 (100)	23 (100)	90 (34–177)		81 (20–259)	
Propionic	17 (71)	18 (78)	5.0 (ND–63)		6.3 (ND–53)	
Butyric	18 (75)	15 (65)	2.9 (ND–37)		1.1 (ND–30)	
Iso-butyric	3 (13)	7 (30)	ND (ND–4.1)		ND (ND–4.0)	
Iso-valeric	6 (25)	7 (30)	ND (ND–3.8)		ND (ND–4.4)	
Valeric	4 (17)	1 (4)	ND (ND–9.8)		ND (ND–0.4)	
Iso-caproic	3 (13)	1 (4)	ND (ND–1.7)		ND (ND–0.3)	
Caproic	4 (17)	1 (4)	ND (ND–1.8)		ND (ND)	
**1 year**^b^						
Total SCFAs	–	–	141 (57–359)		142 (82–253)	
Acetic	28 (100)	36 (100)	87 (44–221)		92 (45–154)	
Propionic	28 (100)	36 (100)	28 (3–48)		31 (8–87)	
Butyric	28 (100)	36 (100)	25 (8–81)		20 (4–59)	
Iso-butyric	22 (79)	29 (81)	1.5 (ND–4.8)		1.4 (ND–5.2)	
Iso-valeric	27 (96)	35 (97)	2.2 (ND–7.6)		2.0 (0.2–6.9)	
Valeric	24 (86)	27 (75)	0.3 (ND–4.8)		0.3 (ND–3.8)	
Iso-caproic	5 (18)	7 (19)	ND (ND–1.2)		ND (ND–0.9)	
Caproic	5 (18)	7 (19)	ND (ND–0–3)		ND (ND–0–3)	
**3 years**^c^						
Total SCFAs	–	–	157 (71–287)		152 (62–283)	
Acetic	19 (100)	32 (100)	83 (46–163)		86 (32–133)	
Propionic	19 (100)	32 (100)	33 (14–49)		30 (8–66)	
Butyric	19 (100)	32 (100)	26 (6–94)		28 (7–70)	
Iso-butyric	19 (100)	29 (91)	2.8* (ND–5.0)		1.6* (ND–5.3)	
Iso-valeric	19 (100)	30 (94)	3.7* (1.3–7.6)		2.4* (ND–8.8)	
Valeric	19 (100)	31 (97)	2.7* (0.4–6.2)		1.7* (ND–4.4)	
Iso-caproic	0 (0)	3 (10)	ND (ND)		ND (ND–0.3)	
Caproic	6 (32)	11 (34)	ND (ND–1.9)		ND (ND–1.3)	

*P < 0.05, Chi-squared or Fisher’s exact test for counts, Mann–Whitney U-test for continuous variables.

ND, not detected. Feces analyzed from: ^a^24 (86%) farm and 23 (62%) control children.

^b^28 (100%) farm and 36 (97%) control children.

^c^19 (68%) farm and 32 (87%) control children.

### Association between life-style factors and SCFA patterns

There were a number of differences in the demographic, family, birth and dietary characteristics of farm and rural control children (Table 1), many of which could influence gut microbiota composition. Multiple logistic regression models were used to determine whether living on a farm independently predicted the SCFA levels at 3 years of age significant in univariate analysis, after adjusting for covariates (Table 3). The models also enabled us to investigate factors that may help explain, and possibly mediate, the distinct SCFA profile seen in farm children, as well as identify factors that predicted SCFA levels in all children. Variables listed in Table 1 were screened for inclusion in the models. In addition, elder siblings and dietary intakes of protein and fibre were included as they have been linked previously with SCFA levels^9,18^.

**Table 3:** Logistic regression models of demographic, family, birth and dietary characteristics on the associations between farm residence and high concentrations of fecal iso-butyric, iso-valeric and valeric acids at 3 years of age.

SCFA^a^	Model type	Predictor variable(s)	OR	(95% CI)	*P*
Iso-butyric	Univariate	Living on a farm	4.7	(1.3–16)	0.02*
	Multivariate^b^	Living on a farm	17	(2.1–131)	0.008**
		Protein (g/day)^c,d^	1.3	(1.1–1.5)	0.002**
		Fibre (g/day)^c,d^	0.5	(0.3–0.8)	0.003**
		Homemade porridge (g/day)^c,e^	1.0	(1.0–1.1)	0.08
Iso-valeric	Univariate	Living on a farm	3.6	(1.0–12)	0.04*
	Multivariate^b^	Living on a farm	6.7	(1.5–30)	0.01*
		Protein (g/day)^c,d^	1.1	(1.0–1.3)	0.01*
		Fibre (g/day)^c,d^	0.7	(0.5–1.0)	0.04*
Valeric	Univariate	Living on a farm	6.9	(1.7–28)	0.008**
	Multivariate^b^	Living on a farm	6.4	(1.4–31)	0.02*
		Siblings	6.2	(1.5–26)	0.01*
		Cat or dog^e^	3.3	(0.8–13)	0.10

*P < 0.05, binary logistic regression, n = 19 (farm) and 32 (control).

^a^SCFA levels dichotomized as concentration of iso-butyric acid, > or ≤ 2.0 μmol/g; iso-valeric acid, > or ≤ 2.5 μmol/g; valeric acid, > or ≤ 2.0 μmol/g.

^b^Final multivariate model. Additional variables related to farm residence and fecal SCFA concentration with *P* < 0.2, and therefore included in the model-building process, were: (iso-butyric acid) intake of homemade porridge and cream; (iso-valeric acid) cream; (valeric acid) father’s education level. Elder siblings and dietary intakes of protein and fibre were also included, as they have been linked theoretically with SCFA levels.

^c^At 1 year of age. We hypothesized that intake at 1 year of age may well be similar to that at 3 years of age and/or may influence the development trajectory of the gut microbiota and short chain fatty acid profile.

^d,e^The primary OR ^(d)^ increased or ^(e)^ decreased by ≥ 10% when the variable was removed and subsequently reintroduced into the model.

After adjustment, living on a farm continued to predict the concentrations of fecal iso-butyric, iso-valeric and valeric acids at 3 years of age (iso-butyric acid, aOR 17, 2.1–131, *P* = 0.008; iso-valeric acid, aOR 6.7, 1.5–30, *P* = 0.01; valeric acid, aOR 6.4, 1.4–31, *P* = 0.02). Further, having elder siblings predicted the concentration of fecal valeric acid independent of the farming effect (aOR 6.2; 95% CI 1.5–26; *P* = 0.01). Living with a cat or dog partly explained the higher concentration of fecal valeric acid in farm children, as indicated by a decrease in the principal odds ratio of 10% on addition of this variable to the logistic regression model (aOR 3.3; 95% CI 0.8–13; *P* = 0.10).

Iso-butyric and iso-valeric acids are produced by microbial fermentation of the amino acids valine and leucine^10^, and valeric acid is likewise generated from the amino acids proline and hydroxyproline^22^. Using data from dietary registration at 1 year of age, we observed associations between dietary protein intake at 1 year of age and the concentration of fecal iso-butyric and iso-valeric acid at 3 years of age (aOR 1.3; 95% CI 1.1–1.5; *P* = 0.002 and aOR 1.1; 95% CI 1.0–1.3; *P* = 0.01, respectively), independent of farm residence. Dietary fibre intake at 1 year of age was negatively associated with the concentration of fecal iso-butyric and iso-valeric acid at 3 years of age (aOR 0.5; 95% CI 0.3–0.8; *P* = 0.003 and aOR 0.7; 95% CI 0.5–1.0; *P* = 0.04, respectively), independent of farm residence. No association was seen between protein or fibre intake and fecal valeric acid. Dietary intake of homemade porridge at 1 year of age partly explained the higher concentration of iso-butyric acid in farm children (aOR 1.0; 95% CI 1.0–1.1; *P* = 0.08).

### Links between SCFA levels and allergy development

We investigated whether fecal SCFAs at 3 years of age were associated with eczema, asthma or allergic rhino-conjunctivitis at 8 years of age. No children had food allergy at 8 years of age. Of the 48 children who were clinically evaluated, nine had no fecal samples available. The analysis therefore included 39 children, nine of whom were allergic and 30 of whom had no allergy and were classified as non-allergic.

**Figure 3:**
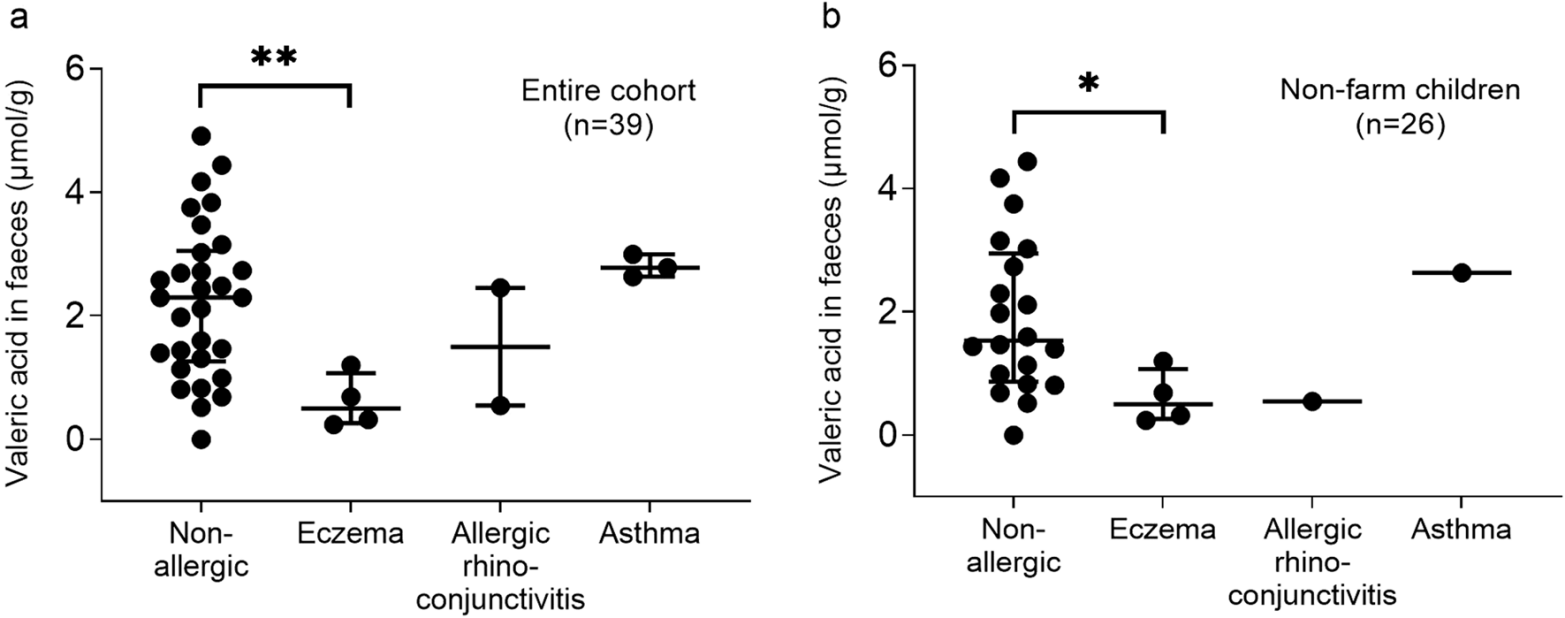
Fecal valeric acid levels at 3 years of age in relation to allergic manifestation at 8 years of age. In (**a**) the whole cohort (n = 39), including both farm and non-farm children, are shown, while (**b**) shows control children only (n = 26). Non-allergic children were defined as having no allergy at 8 years of age. *P < 0.05, **P < 0.01, Mann–Whitney U-test. Vertical bars show the median and inter-quartile range.

Children with eczema at 8 years of age had a lower median concentration of valeric acid in their feces at 3 years of age than non-allergic children (0.5 vs. 2.3 μmol/g, *P* = 0.007; Fig. 3a). Other allergic manifestations could not be linked to valeric acid concentration (Fig. 3a), and concentrations of other SCFAs were not related to any allergy diagnosis at 8 years of age.

An analysis of only control children, to avoid possible confounding by farm residence, included just six allergic and 20 non-allergic children. Again, children who had eczema at 8 years of age had a lower median concentration of fecal valeric acid at 3 years of age than non-allergic children (0.5 vs. 1.5 μmol/g, *P* = 0.03; Fig. 3b).

**Figure 4:**
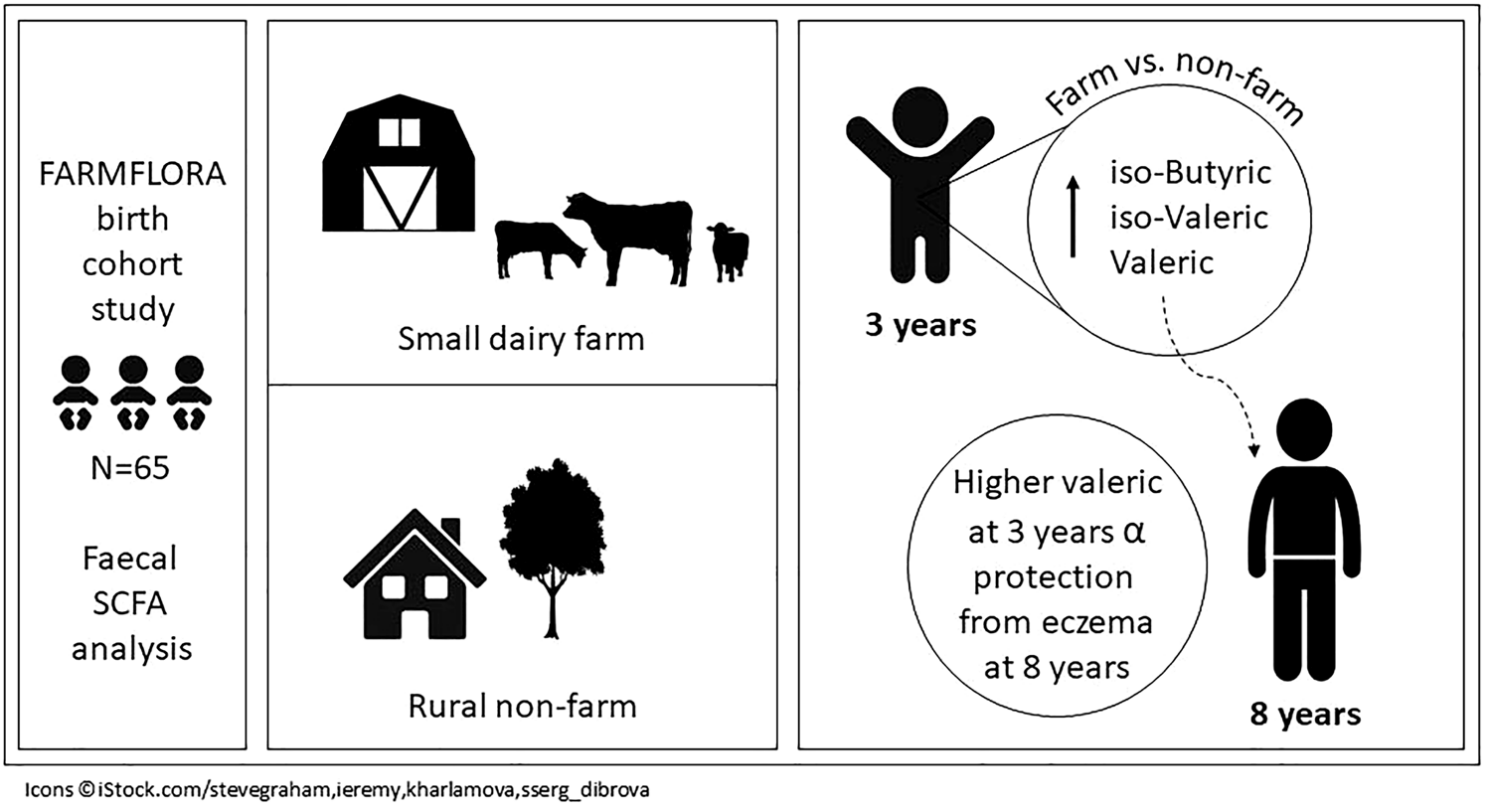
Overview of key findings.

An overview of key findings is presented in Fig. 4.

## Discussion

Short chain fatty acids are end-products of fermentation of carbohydrate and protein by bacteria in the colon, and the pattern of individual SCFAs depends on microbiota composition, and also on the availability of substrates for fermentation. In early childhood, a gradual development of a more and more complex gut microbiota is seen, from a rather simple composition in early infancy, with the successive establishment of more and more strict anaerobes leading to a pronounced dominance of anaerobic bacteria at 2 or 3 years of age^23^.

In the early months, the major end points of metabolism of the gut microbes are acetic and lactic acid. Lactic acid is non-volatile and not detected by gas chromatography. Thus, acetic acid is by far the most abundant SCFA measured in the colon. The trends we observed in SCFA level from 4 weeks to 3 years of age, namely an early dominance of acetic acid (C_2_) followed by the progressive increase in levels of the longer (C_3–5_) and branched (*i*C_4–5_) SCFAs, are consistent with findings from a number of previous studies^18,24,25^. In an adult-pattern microflora containing hundreds of different species^26^, different groups of bacteria species possess specific enzymes involved in the production of the various SCFAs^9^. Indeed, the longest (C_5–6_) and branched SCFAs are produced by anaerobes belonging to the dominant *Bacteroidetes* and *Firmicutes* phyla, often via cross-feeding pathways^27–29^.

Iso-butyric and iso-valeric acids are produced via the microbial fermentation of the amino acids valine and leucine^10^. Valeric acid is likewise generated from the amino acids proline and hydroxyproline^22^, as well as by the microbial metabolism of lactate^30^ and propionate^31^. Accordingly, dietary protein intake at 1 year of age predicted levels of fecal iso-butyric and iso-valeric acid at 3 years of age. We have earlier reported quite pronounced differences in dietary pattern between farming and non-farming families in our cohort, regarding the maternal diet during pregnancy^32^, fatty acid pattern in maternal breastmilk^32^, and the children’s diet at 12 months of age^8^, with intake of more full-fat dairy products and saturated fat in the farm mothers and children. However, protein intake did not vary between farm and control children in this study. Multiple logistic regression models confirmed that differences in diet and other factors we collected data on did not account for the increased levels of iso-butyric, iso-valeric and valeric acids observed at 3 years of age in farm children compared to controls in this study. Our results accordingly suggest that a more mature, complex gut microbiota in the farm children is the chief explanation for the increased levels of these SCFAs.

We observed substantially lower concentrations of fecal valeric acid at 3 years of age in children who had eczema at 8 years of age, as compared to non-allergic children. This relationship was evident in our entire cohort, and also in a sub-analysis of control children, indicating that high levels of fecal valeric acid derived from non-farm exposures are also associated with protection from eczema, as discussed more fully below. However, levels of fecal valeric acid were higher in farm children than in non-allergic controls (median 2.7 vs. 1.5 μmol/g, *P* = 0.04), in line with the particularly strong degree of allergy protection conferred by growing up on a farm. We could not link valeric acid levels with allergic rhino-conjunctivitis or asthma, but these categories contained few children. Also, we did not find any association between the concentration of iso-butyric or iso-valeric acid at 3 years of age and any of the allergic conditions we assessed for at 8 years of age. Our findings suggest that farm-related protection from allergy may be mediated in part by fecal valeric acid, or by characteristics of the gut microbiota associated with fecal valeric acid.

To date, few other prospective studies have described fecal SCFA patterns in relation to subsequent allergy. However, consistent with our findings, Sandin et al. observed lower levels of fecal iso-butyric, iso-valeric and valeric acid at 1 year of age in children with reported food allergy at 4 years of age in the BarnAllergiStudie (BAS) birth cohort study^18^. Further, data from the same birth cohort show lower levels of fecal valeric acid at 1 year of age in children with eczema, and also food allergy, at 13 years of age (Gio-Batta et al., Low levels of fecal valeric acid at 1 year of age are associated with eczema and food allergy at 13 years of age in a prospective birth cohort). Other prospective studies have generally not included valeric acid in the SCFA analysis. However, in another birth cohort, lower levels of fecal propionate and butyrate at 12 months of age were linked with higher risk of sensitization between 3 and 6 years of age^33^. Further, in a clinical trial of non-digestible oligosaccharides in infant formula in high-risk infants, higher levels of fecal propionate and butyrate at 3 months of age, but lower levels of the same SCFAs at 6 months of age, were associated with eczema at 18 months^34^.

The pathogenesis of allergy has variously been related to impairment of epithelial barrier function, altered immune responses and dysbiosis of the gut microbiota^35–37^. Recent studies demonstrate that valeric acid upregulates tight junction proteins and also keratin (KRT1), promoting tissue integrity and barrier function^38,39^. Conversely, KRT1-deficient mice show impaired skin barrier function and have a gene expression signature similar to that observed in skin of human eczema patients^40^. Valeric acid also induces IL-10 production, inhibits IL-17 and increases survival of regulatory B cells, thereby promoting the regulatory activity of lymphocytes^41,42^. In addition, valeric acid is antimicrobial^43^ and oral administration of valeric acid esters to rodents either fed a high fat diet or subjected to irradiation helps protect against gut dysbiosis^39,44^. Further, administration of valeric acid or its esters to animal models protects against colitis^39^ and necrotic enteritis^45^, while adoptive transfer of valerate-treated Bregs together with naive CD4 + T cells into Rag1-deficient mice protects against colitis and experimental autoimmune encephalitis^41^. These studies suggest multiple mechanisms by which valeric acid may potentially protect against allergy, and show in-vivo efficacy of valeric acid against other immune-mediated diseases.

Besides farm residence and diet, some other factors also affected SCFA pattern. These included living with a cat or dog, which helped explain the increased fecal valeric acid levels in children raised on farms, as they were more likely to own a pet than control children. Accordingly, data regarding the microbial composition of this cohort show a larger influence of cats and dogs on the gut microbiota than farm residence itself (Ljung et al., Effects of pets and farm residence on gut microbiota establishment and implications for allergy development). We also observed that children with older siblings had higher levels of fecal valeric acid, as was also noted by Sandin et al.^18^. Interestingly, infants with no older siblings tend to have an impoverished microbiota compared to infants with siblings^46^. Both elder siblings and early pet exposure have also been associated with reduced risk of allergic disease^47,48^.

The major limitation of this study was the moderate number of study subjects, yielding a limited number of allergic children. The strengths were the longitudinal design, permitting analysis of SCFA pattern development during early childhood and measurement of a broad range of dietary and other exposure data, as well as allergy being stringently diagnosed by study pediatricians. However, no environmental microbial samples were collected, and therefore the hypothesis that increased microbial exposure on farms may affect the structure and function of the gut microbiota could not be addressed directly.

In conclusion, growing up on a small dairy farm is associated with a fecal SCFA pattern suggestive of a more mature and complex anaerobic gut microbiota at 3 years of age, as compared to living in the surrounding rural area but not on a farm. Specifically, farm children had higher levels of fecal iso-butyric, iso-valeric and valeric acids. Diet could not explain this pattern, but exposure to pets accounted for part of the farming effect. High levels of fecal valeric acid were inversely associated with eczema at 8 years of age, suggesting that valeric acid may explain part of the farm-related protection from allergy. Although this finding needs to be confirmed in larger studies, it implies that valeric acid or microbes producing this metabolite could potentially offer therapeutic potential in preventing or treating eczema.

## Data Availability

The datasets generated during and/or analysed during the current study are available from the corresponding author on reasonable request.
